# Foundation Protein Language Models for Influenza A Virus T-Cell Epitope Prediction: A Transformer-Based Viroinformatics Framework

**DOI:** 10.3390/v18030380

**Published:** 2026-03-18

**Authors:** Syed Nisar Hussain Bukhari, Kingsley A. Ogudo

**Affiliations:** 1 National Institute of Electronics and Information Technology (NIELIT) J&K, Srinagar 191132, India; 2 Department of Electrical & Electronic Engineering Technology, University of Johannesburg, Johannesburg 2094, South Africa; kingsleyo@uj.ac.za

**Keywords:** attention mechanisms, Influenza A virus, protein language models, T-Cell epitope, transformer networks, uncertainty estimation

## Abstract

Influenza A virus remains a major cause of respiratory disease worldwide and poses a persistent challenge to vaccine development due to its rapid genetic evolution and antigenic variability. T-cell-based immunity has therefore gained increasing importance, as it can provide broader and more durable protection by targeting conserved viral regions. Accurate identification of T-cell epitopes (TCEs) is a fundamental requirement for epitope-based vaccine design and immunological research. Although numerous computational methods have been proposed, many existing approaches rely on handcrafted physicochemical features, which offer limited ability to capture contextual sequence dependencies. In this study, a transformer-based viroinformatics framework is proposed for the binary prediction of TCEs from Influenza A virus peptide sequences. The framework employs a pretrained Evolutionary Scale Modeling-2 (ESM-2) protein language model (PLM) to generate rich, contextualized embeddings directly from raw amino acid sequences, eliminating the need for manual feature engineering. These embeddings are processed using a lightweight attention-based transformer classifier to learn epitope-specific sequence patterns. The model achieves strong and stable predictive performance, attaining an accuracy of approximately 97% and an AUC close to 0.99 under stratified cross-validation. Ablation analysis further confirms that protein language model representations and self-attention contribute substantially to performance gains over classical machine learning baselines. To enhance practical reliability, Monte Carlo dropout is incorporated during inference to provide uncertainty-aware predictions, enabling differentiation between high-confidence and ambiguous peptide candidates. In addition, attention-based interpretability is used to identify residue-level contributions to model decisions, offering biologically meaningful insights into epitope recognition. Overall, this study demonstrates that PLMs combined with Transformer architectures provide an effective, interpretable, and a promising computational framework for Influenza A TCE discovery and vaccine research.

## 1. Introduction

Influenza viruses are segmented, negative-sense ribonucleic acid (RNA) viruses belonging to the Orthomyxoviridae family and are responsible for recurrent respiratory infections in humans and animals [1]. Based on antigenic differences in the nucleoprotein and matrix protein, influenza viruses are classified into three major types: A, B, and C. Among these, Influenza A virus is the most clinically significant due to its broad host range, high transmissibility, and ability to cause severe disease outbreaks [2]. Seasonal influenza epidemics and periodic pandemics associated with Influenza A virus continue to result in substantial morbidity and mortality worldwide, making it a persistent public health concern.

A defining characteristic of Influenza A virus is its pronounced genetic variability. The virus evolves rapidly through molecular mechanisms such as antigenic drift, driven by point mutations, and antigenic shift, resulting from reassortment of gene segments when multiple viral strains co-infect the same host [3]. These evolutionary processes can give rise to novel viral subtypes with altered antigenic profiles, as observed during historical influenza pandemics. Such genetic plasticity frequently leads to a mismatch between circulating viral strains and existing vaccines, thereby reducing vaccine effectiveness and necessitating continuous surveillance and vaccine reformulation [4]. Given these challenges, the development of broadly protective and long-lasting vaccines remains a critical objective in influenza research. In recent years, increasing attention has been directed toward cell-mediated immunity, particularly T-cell-based responses, which have been shown to provide cross-strain protection by recognizing conserved viral regions [5]. Central to this approach is the identification of T-cell epitopes (TCEs), short peptide fragments derived from viral proteins that are presented by major histocompatibility complex (MHC) molecules and recognized by T lymphocytes [6]. However, the experimental identification of TCEs through immunological assays is labor-intensive, costly, and difficult to scale across the full viral proteome [7]. As a result, computational methods have emerged as indispensable tools for accelerating epitope discovery and guiding experimental validation [8]. These methods aim to rapidly screen large numbers of peptide sequences and prioritize candidates for downstream experimental validation, thereby reducing both cost and development time [3].

Traditional computational approaches to epitope prediction have largely relied on handcrafted physicochemical features, such as amino acid composition, hydrophobicity indices, charge, and sequence-derived descriptors. While these features are biologically motivated and have shown utility in various machine learning (ML) frameworks, they suffer from inherent limitations [9]. Most notably, such representations treat residues in a largely independent or weakly contextual manner and depend heavily on prior assumptions about which properties are most relevant [10]. This limits their ability to capture complex sequence dependencies and often reduces generalization when applied to unseen peptides or evolving viral strains [11].

Recent advances in deep learning (DL) have introduced new opportunities to address these limitations. In particular, protein language models (PLMs), inspired by natural language processing, have demonstrated the ability to learn rich and contextual representations directly from raw protein sequences [12]. Trained on large-scale protein databases using self-supervised objectives, these models capture sequence patterns, residue relationships, and evolutionary constraints without requiring manual feature engineering [13]. When combined with attention-based architectures such as Transformers, these representations enable models to reason about interactions across entire sequences, making them especially suitable for tasks involving short but information-dense peptides [14]. Recent work has also explored adapting pretrained protein language models to specialized biological domains, including immunological sequence analysis, further highlighting their potential for epitope prediction and related immunoinformatics applications.

Recent advances in immunoinformatics have also introduced a number of DL–based frameworks for epitope prediction. Convolutional neural network (CNN) architectures have been widely explored for modeling sequence patterns associated with immunogenic peptides, demonstrating improved performance over traditional machine learning models that rely on handcrafted descriptors. More recently, hybrid architectures integrating multiple deep learning components have been proposed. For example, the MITNet framework combines convolutional layers with Transformer-based attention mechanisms to capture contextual relationships within peptide sequences [13]. These studies highlight the increasing use of deep neural networks to model complex sequence dependencies relevant to immune recognition.

In this study, a transformer-based viroinformatics framework is proposed for the prediction of TCEs from Influenza A virus peptide sequences [15,16,17]. The framework leverages a pretrained PLM to generate contextualized sequence embeddings that serve as an automated and biologically informed alternative to handcrafted features [18]. Among available protein language models, Evolutionary Scale Modeling-2 (ESM-2) was selected due to its strong representational capability, compact architecture, and suitability for modeling short peptide sequences, making it well aligned with TCE prediction tasks [19]. These embeddings are subsequently processed by a lightweight Transformer classifier that learns epitope-specific patterns through self-attention [20]. To enhance practical utility, the framework incorporates uncertainty-aware prediction using Monte Carlo dropout, allowing the model to associate confidence estimates with its predictions [21]. In addition, attention-based analysis is employed to provide residue-level interpretability, offering insight into sequence regions that influence model decisions.

The proposed approach is evaluated within a binary classification setting, distinguishing epitopes from non-epitopes, and is assessed using a comprehensive set of performance metrics and validation strategies [22,23]. Comparative and ablation analyses are conducted to examine the contribution of individual architectural components and to contextualize performance relative to existing methods [24].

The main contributions of this study can be summarized as follows:

1. A protein language model-based framework is introduced for TCE prediction that eliminates the need for manual physicochemical feature engineering.
2. A task-specific Transformer classifier is employed to capture residue-level interactions relevant to epitope recognition.
3. An uncertainty-aware prediction mechanism is integrated to distinguish high-confidence predictions from ambiguous cases.
4. Attention-based interpretability is provided to support biological insight and transparency in model behavior.
5. The framework is systematically evaluated on Influenza A virus peptide sequences, demonstrating stable and reliable performance.

## 2. Materials and Methods

**Figure 1:**
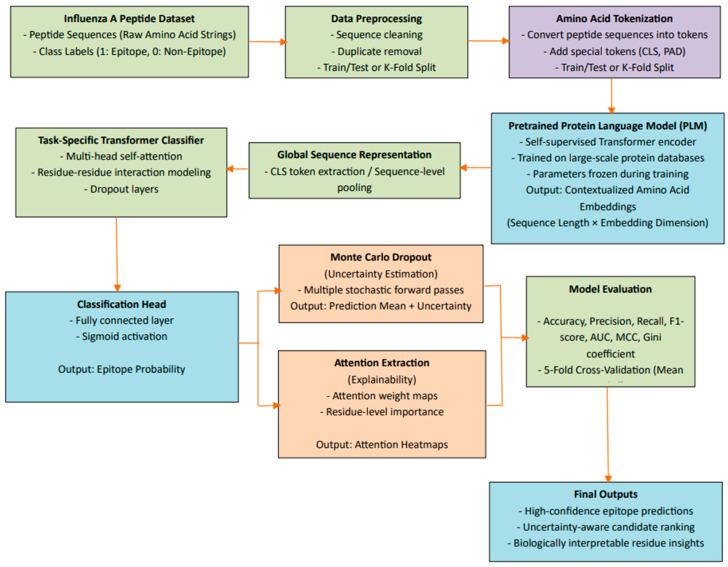
Overview of the proposed protein language model–driven Transformer framework for Influenza A T-cell epitope prediction. Peptide sequences are first processed by the pretrained ESM-2 model to obtain contextual embeddings, which are then analyzed by a lightweight Transformer classifier to model residue-level interactions and predict epitope probability. Monte Carlo dropout is incorporated for uncertainty estimation, while attention analysis provides interpretability of residue contributions.

The overall workflow of the proposed transformer-based viroinformatics framework is illustrated in Figure 1. Each component of the framework is described in detail in the following subsections.

### 2.1. Acquiring Dataset

The dataset used in this study consists of experimentally validated peptide sequences derived from Influenza A virus proteins from the immune epitope database (IEDB) [25]. Each peptide is annotated as either a TCE or a non-epitope, forming a binary classification dataset. The annotations are based on curated experimental evidence reported in publicly available immunological repositories. All peptide sequences were subjected to a standard curation pipeline prior to model development. Duplicate sequences were removed to avoid information leakage between training and evaluation phases [26]. Because the peptide sequences analyzed in this study are relatively short (8–15 amino acids), additional clustering by sequence identity was not applied in order to preserve dataset diversity and avoid excessive reduction in available samples. Peptides containing ambiguous or non-standard amino acid symbols were excluded to preserve biological validity. No synthetic data generation, resampling, or class balancing strategies were applied, as the dataset exhibited an approximately balanced distribution of epitopes and non-epitopes. Peptide lengths spanned a biologically meaningful range consistent with known TCE characteristics. For model evaluation, stratified sampling strategies were employed to preserve the class distribution across training and validation subsets. In addition to fixed train-test splits, stratified cross-validation was used to assess robustness and reduce sensitivity to data partitioning.

### 2.2. Problem Formulation and Learning Objective

The epitope prediction task is formulated as a binary sequence classification problem. Given an input peptide sequence $s=(a_{1},a_{2},\ldots ,a_{n})$, where $a_{i}$ represents an amino acid residue, the objective is to learn a function:

f(s)→{0,1}

where 1 denotes a T-cell epitope and 0 denotes a non-epitope.

Rather than producing a hard class label directly, the model outputs a probability score in the range $\left[0,1\right]$, representing the likelihood that a given peptide is an epitope. This probabilistic formulation enables downstream confidence analysis and uncertainty estimation while retaining a binary decision boundary for evaluation. For evaluation purposes, a default probability threshold of 0.5 was used to convert predicted probabilities into binary epitope or non-epitope class labels.

### 2.3. Protein Language Model-Based Representation Learning

Protein Language Models (PLMs) form the representational foundation of the proposed framework. These models are trained on large collections of protein sequences using self-supervised objectives, allowing them to learn statistical and structural regularities inherent to biological sequences [18]. Through this process, PLMs capture residue compatibility, sequence context, and evolutionary constraints without relying on manually defined biochemical features.

In this study, the Evolutionary Scale Modeling, version 2 (ESM-2) PLM was employed as the pretrained PLM backbone [19]. ESM-2 is a Transformer-based model trained on large-scale protein sequence corpora using self-supervised learning objectives, enabling it to capture rich contextual and evolutionary information from amino acid sequences. Compared to earlier protein embedding approaches, ESM-2 provides compact yet expressive representations that are well suited for modeling short peptide sequences. In the proposed framework, ESM-2 is used strictly as a frozen feature extractor, and no task-specific fine-tuning of the language model parameters is performed. The pretrained PLM is used exclusively as an automated feature extractor wherein each peptide sequence is tokenized at the amino acid level and passed through the PLM, which produces a high-dimensional embedding for every residue. These embeddings are contextualized, meaning each residue representation depends on its surrounding amino acids rather than its identity alone.

To enable downstream classification, a fixed-length representation is derived from the sequence-level embedding (CLS token) [27]. This vector acts as a compact summary of the entire peptide, capturing both local residue properties and global sequence patterns. The PLM parameters are kept frozen during training to retain general protein knowledge, reduce overfitting, and improve computational efficiency. The role of the PLM within the overall pipeline is illustrated in the model architecture diagram.

### 2.4. Transformer-Based Epitope Classification Module

While the PLM provides biologically informed sequence representations, it is not trained to discriminate epitopes from non-epitopes. To address this, a task-specific Transformer-based classifier is employed [16]. The Transformer architecture leverages self-attention to model interactions between different residue positions within a peptide, allowing the classifier to focus on residue combinations that are informative for epitope recognition.

The Transformer module used in this framework is a lightweight, task-specific encoder designed to operate on PLM-derived sequence representations rather than raw amino acid tokens. Unlike the pretrained Transformer architecture underlying the protein language model, this classifier is trained from scratch and optimized exclusively for the epitope prediction task [17]. Its role is to refine the contextual embeddings produced by the PLM by learning epitope-relevant residue interactions through self-attention, thereby bridging general protein sequence knowledge and task-specific discrimination.

Self-attention allows the model to weigh residues according to their contribution to the classification decision. This is particularly relevant for epitope prediction, where immunogenicity often depends on motifs or positional relationships rather than on individual residues in isolation. Multi-head attention further allows the model to capture multiple interaction patterns simultaneously. The output of the Transformer encoder is passed through a fully connected layer with a sigmoid activation function, producing a probability score that reflects epitope likelihood. The complete computational workflow of the model is formally summarized in Algorithm 1.


**Algorithm 1:** PLM-Transformer-Based T-Cell Epitope Prediction**Input:** Peptide dataset $D=\{(s_{i},y_{i}){\}}_{i=1}^{N}$ where $s_{i}$ is a peptide sequence and $y_{i}\in \{0,1\}$ **Output:** Predicted epitope probability ${\hat{y}}_{i}$, uncertainty $u_{i}$ 1:  Initialize pretrained Protein Language Model (PLM) 2:  Freeze PLM parameters 3:  Initialize Transformer-based classifier T 4:  Define loss function L and optimizer O 5:  Perform K-fold cross-validation 6:  for k = 1 to K do 7: Split D into training set Dtrain and test set Dtest 8: for epoch = 1 to E do 9:  for each mini-batch (S, Y) in Dtrain do 10:   Tokenize peptide sequences S 11:   E ← PLM(S)        # contextual embeddings 12:   Z ← T(E)        #attention-based encoding 13:   Ŷ ← Sigmoid(Z)    #prediction probabilities 14:   Compute loss L(Ŷ, Y) 15:   Update T using optimizer O 16:  end for 17: end for 18: Evaluate T on Dtest and store performance metrics 19: end for 20: Compute mean and standard deviation of metrics across folds 21: Enable dropout layers in T 22: for each peptide s in D do 23: Perform multiple stochastic forward passes 24: Compute mean prediction ŷ and uncertainty u 25: end for 26: Return predictions, uncertainties, and performance statistics


### 2.5. Model Training Strategy and Optimization

Model training is restricted to the parameters of the Transformer-based classifier, while the PLM remains frozen [15]. This separation allows the model to exploit pretrained biological knowledge while learning task-specific decision boundaries from labeled epitope data. Training uses mini-batch gradient descent optimized with binary cross-entropy loss, which measures the discrepancy between predicted probabilities and true labels.

An adaptive optimization algorithm is used to handle variability in peptide sequence patterns. Dropout layers are incorporated within the Transformer encoder as a regularization mechanism to mitigate overfitting. These dropout layers also serve a secondary role during inference for uncertainty estimation.

### 2.6. Uncertainty-Aware Prediction Using Monte Carlo Dropout

To quantify prediction confidence, the proposed framework incorporates Monte Carlo dropout. During inference, dropout remains active and multiple stochastic forward passes are performed for each peptide sequence [21]. The mean prediction across these passes is used as the final epitope probability, while the variability across predictions is interpreted as predictive uncertainty.

Low uncertainty indicates stable model behavior and high confidence in the classification outcome, whereas higher uncertainty highlights ambiguous peptides that may require further experimental validation. This uncertainty-aware formulation is particularly valuable in immunological applications, where computational predictions are often used to prioritize candidates for downstream laboratory testing.

### 2.7. Model Interpretability via Attention Analysis

To improve transparency, the self-attention weights learned by the Transformer classifier are extracted and analyzed. These weights reflect how strongly different residue positions influence one another during classification. Visualizing attention distributions helps identify residues or regions that contribute strongly to the model’s decision [28].

Although attention weights do not establish causal relationships, they provide useful qualitative insight into model focus and allow assessment of whether predictions are driven by biologically plausible sequence regions [29]. This interpretability layer helps bridge the gap between DL predictions and immunological reasoning.

### 2.8. Performance Evaluation Protocol

Model performance is evaluated using a comprehensive set of binary classification metrics, including accuracy, precision, recall (sensitivity), specificity, F1-score, area under the Receiver Operating Characteristic (ROC) curve, Matthew’s correlation coefficient, and Gini coefficient [30]. These metrics jointly capture predictive accuracy, class balance, and discrimination capability.

To assess robustness, stratified cross-validation is employed, ensuring that each fold maintains the original class distribution. Confusion matrix analysis is used to examine error patterns, while ROC analysis evaluates threshold-independent discrimination performance. Together, these evaluation strategies provide a rigorous assessment of model reliability.

### 2.9. Comparative Evaluation and Ablation Analysis

The proposed framework is evaluated against classical machine learning models trained on handcrafted physicochemical features to assess the benefit of representation learning. Additional comparisons with existing epitope prediction tools and DL baselines without pretrained embeddings are conducted to contextualize performance gains. Ablation experiments are performed by selectively removing or modifying key components, such as the protein language model or the attention mechanism [31]. These experiments isolate the contribution of each component and clarify how architectural design choices influence predictive behavior.

### 2.10. Implementation and Computational Environment

All experiments are implemented using Python (version 3.12)-based ML libraries. DL components are developed using PyTorch (version 2.7.0), while pretrained protein language models are integrated through transformer-based frameworks [32]. Evaluation procedures are implemented using standard scientific computing tools. Experiments are conducted on hardware equipped with Graphics Processing Unit (GPU) acceleration. Freezing the PLM parameters significantly reduces training time and memory requirements, making the framework suitable for practical deployment.

## 3. Results

### 3.1. Dataset Characteristics and Hyperparameter Tuning

The proposed PLM-Transformer framework was evaluated on a comprehensive Influenza A virus peptide dataset curated from IEDB, consisting of 8271 experimentally validated peptide sequences with a near-balanced distribution of epitopes and non-epitopes (Table 1) [25]. The balanced nature of the dataset ensured that performance metrics were not biased toward a dominant class and allowed a reliable assessment of both sensitivity and specificity. Model hyperparameters and architectural configurations used across all experiments are summarized in Table 2, ensuring reproducibility and transparency.

### 3.2. Overall Predictive Performance and Cross-Validation Stability

The predictive performance of the proposed model was primarily assessed using stratified 5-fold cross-validation (5FCV) to evaluate robustness and generalization [34]. As reported in Table 3, the proposed PLM-Transformer model achieved a mean accuracy of 96.84% with low variance across folds, demonstrating strong stability. High sensitivity (97.32%) indicates the model’s ability to correctly identify immunogenic TCEs, while the corresponding specificity (96.36%) confirms reliable discrimination against non-epitopes.

The high AUC value of 0.989 further reflects excellent ranking capability, suggesting that the model consistently assigns higher probabilities to true epitopes than to non-epitopes across decision thresholds. The strong MCC and Gini coefficient values highlight balanced performance even under class-agnostic evaluation criteria, reinforcing the robustness of the proposed framework.

**Figure 2:**
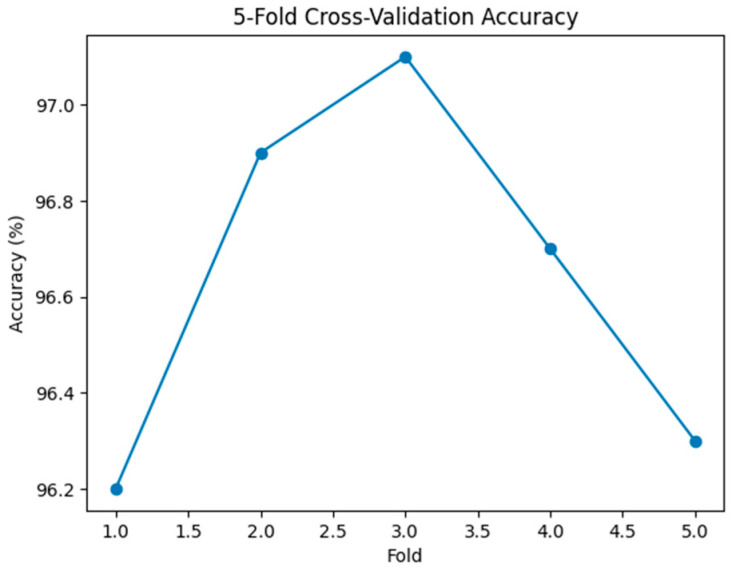
Accuracy distribution across five cross-validation folds.

The consistency of performance across folds is visually illustrated in Figure 2, where minimal fluctuation in accuracy is observed, confirming the absence of split-dependent bias.

### 3.3. Comparison with Classical ML Models and Existing Tools

To contextualize the performance gains, the proposed framework was compared against classical ML models and existing immunoinformatics tools. As shown in Table 4, the PLM-Transformer model substantially outperformed traditional classifiers such as decision trees, SVMs, and random forests, as well as earlier ensemble-based approaches relying on handcrafted physicochemical features. All comparative models were evaluated using the same dataset and evaluation protocol as the proposed framework to ensure fair comparison across methods.

Furthermore, comparison with established epitope prediction tools, including NetMHC and CTLpred (Table 5), demonstrates a clear margin of improvement in accuracy, sensitivity, and specificity. These results indicate that automated representation learning through protein language models, combined with attention-based modeling, captures biologically relevant sequence patterns that are not adequately represented by conventional feature-engineering-driven methods. To ensure a consistent comparison, all evaluated methods were assessed using the same peptide dataset and evaluation metrics, although some existing tools operate under different modeling assumptions such as allele-specific prediction.

**Figure 3:**
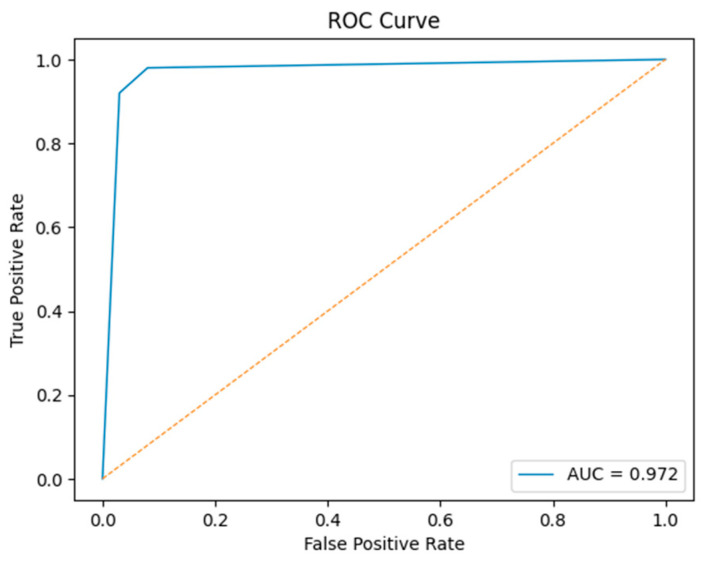
ROC curve of the proposed PLM-Transformer model for Influenza A TCE prediction.

The discriminative capability of the proposed model is further illustrated by the ROC curve in Figure 3, which shows a steep rise toward the top-left corner and confirms near-optimal classification behavior.

### 3.4. Ablation Study and Architectural Contribution Analysis

An ablation study was conducted to quantify the individual contributions of key architectural components [31]. As summarized in Table 6, removing the PLM and reverting to handcrafted physicochemical features resulted in a substantial performance drop, underscoring the critical role of PLM-based representations. While the introduction of a Transformer on top of handcrafted features improved performance, the best results were achieved only when PLM embeddings were combined with attention-based modeling. These findings confirm that performance gains arise not merely from architectural complexity but from the synergy between biologically informed sequence embeddings and task-specific attention learning.

### 3.5. Effect of Attention Configuration

The influence of attention configuration was systematically evaluated by varying the number of attention heads in the Transformer encoder. As shown in Table 7, performance improved as the number of attention heads increased from one to four, reflecting enhanced modeling of residue-residue interactions. Beyond four heads, performance gains saturated while computational complexity increased. Consequently, four attention heads were selected as the optimal configuration, balancing predictive performance and efficiency.

### 3.6. Computational Efficiency and Training Cost

Practical feasibility is an important consideration for large-scale immunoinformatics applications. Table 8 compares the training cost and computational complexity of the proposed model with classical and DL baselines. Despite incorporating a deep representation model, the proposed framework remains computationally efficient due to the use of a frozen PLM and a lightweight Transformer classifier. This design allows the model to achieve competitive predictive performance without prohibitive training overhead, making it suitable for real-world vaccine discovery pipelines.

### 3.7. Epitope Length-Wise Performance Analysis

To assess biological consistency, model performance was analyzed across different peptide length categories. As reported in Table 9, the model exhibited strongest performance for peptides of length 9–11 amino acids, which aligns well with canonical MHC class I binding preferences [40]. Slightly reduced performance for longer peptides is biologically expected due to increased sequence variability and conformational diversity. These results indicate that the model not only performs well statistically but also conforms to established immunological principles.

### 3.8. Explainability and Uncertainty-Aware Analysis

Beyond predictive accuracy, interpretability and reliability are critical for downstream biological validation. The uncertainty-aware analysis using Monte Carlo dropout (Table 10) demonstrates that high-confidence epitope predictions are associated with low predictive uncertainty, whereas borderline peptides exhibit higher uncertainty values [21]. This property is particularly valuable for prioritizing candidates for experimental validation.

The uncertainty estimates provided by Monte Carlo dropout are intended to offer a practical indication of prediction confidence, while more comprehensive calibration or error-correlation analyses remain an important direction for future work.

**Figure 4:**
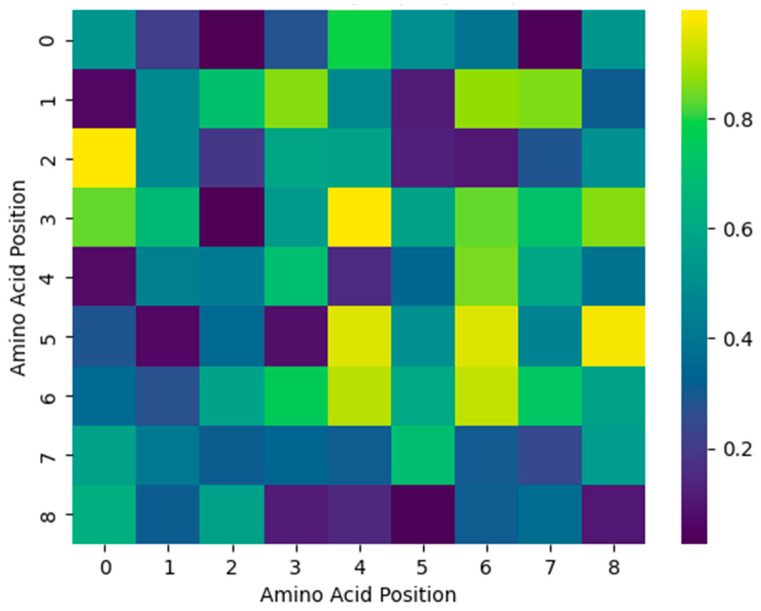
Residue-level attention heatmap highlighting immunodominant positions within an Influenza A epitope peptide.

Model explainability was further investigated through attention-based visualization. Figure 4 illustrates residue-level attention heatmaps for representative Influenza A epitopes, highlighting immunodominant positions that contribute most strongly to the prediction. The example shown in Figure 4 illustrates a representative case highlighting residue-level attention patterns learned by the model. These attention patterns provide biologically meaningful insights into residue importance and motif-like structures.

**Figure 5:**
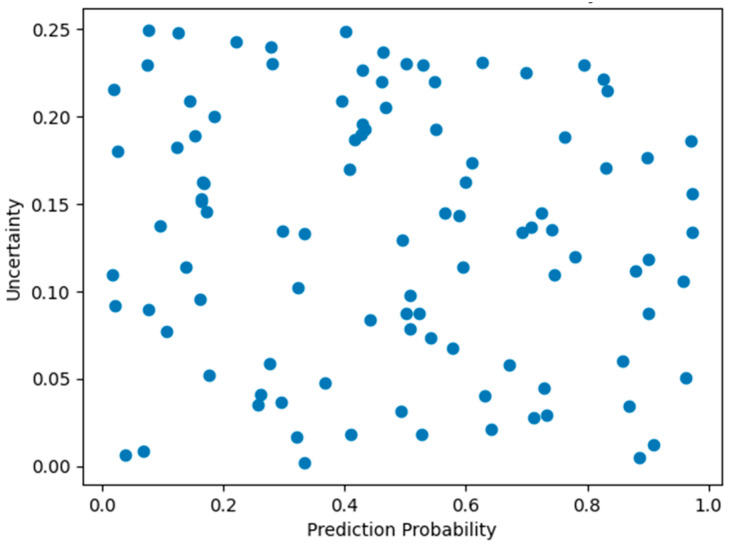
Relationship between predicted probability and uncertainty for test peptides.

**Figure 6:**
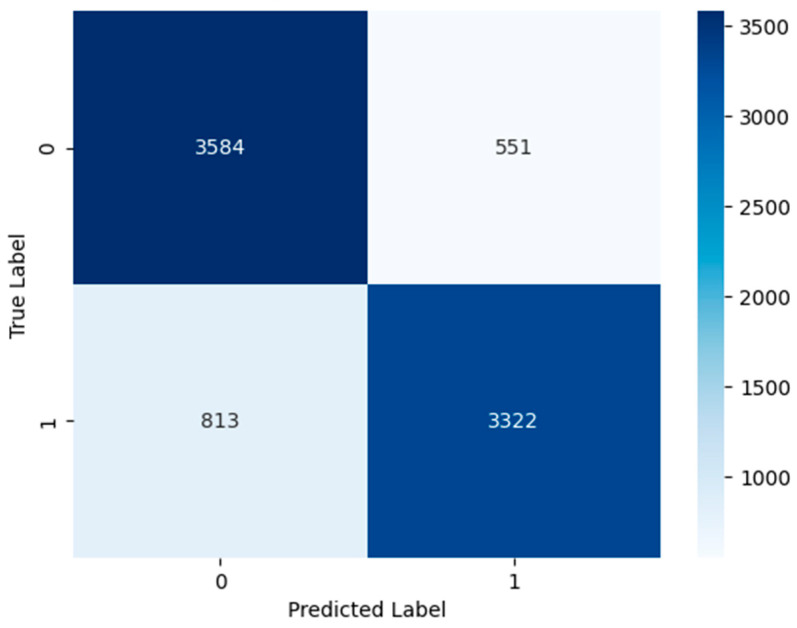
Confusion matrix illustrating the classification outcomes obtained from the evaluation procedure described in this study, showing the distribution of correctly and incorrectly predicted epitope and non-epitope peptides.

Additionally, the relationship between prediction confidence and uncertainty is visualized in Figure 5, where high-confidence predictions cluster at low uncertainty levels, reinforcing the reliability of the proposed framework. The confusion matrix shown in Figure 6 further confirms balanced error distribution between classes, supporting the overall robustness of the model.

## 4. Discussion

This study examined the application of PLMs combined with attention-based Transformer architectures for the binary prediction of TCEs from Influenza A virus peptide sequences [3]. Influenza A virus is characterized by rapid genetic evolution driven by antigenic drift and reassortment, which frequently undermines vaccine efficacy and complicates immune targeting [41]. In this context, computational identification of conserved and immunologically relevant peptide regions represents an important step toward supporting T-cell-based vaccine strategies [13]. Unlike traditional epitope prediction approaches that rely on handcrafted physicochemical descriptors, the proposed framework learns representations directly from raw amino acid sequences, enabling the model to capture contextual and relational information that is difficult to encode manually. The observed performance indicates that this representation-driven strategy offers both reliable predictive capability and improved interpretability.

A key observation from this work is the importance of sequence context in TCE recognition. For Influenza A virus, subtle sequence variations often arising from mutation or reassortment can significantly alter immune recognition while preserving overall protein composition. Classical ML models typically summarize peptides using averaged residue properties or frequency-based descriptors, which tend to overlook such contextual dependencies. In contrast, the PLM employed in this study generates embeddings in which each residue representation is conditioned on its surrounding sequence. This allows the downstream Transformer classifier to model residue interactions and motif-like patterns that are likely to be relevant for T-cell recognition of influenza-derived peptides.

The architectural separation between the pretrained PLM and the task-specific Transformer classifier proved to be an effective design choice. The language model contributes general protein sequence knowledge learned from large-scale pretraining, capturing biologically meaningful patterns that extend beyond virus-specific datasets. The Transformer classifier, in turn, adapts these representations to the epitope prediction task by learning decision boundaries specific to Influenza A virus peptides. Freezing the language model parameters reduced computational cost and limited overfitting, while preserving representational richness. This modular design also provides a flexible computational framework that may be adaptable to other viral pathogens; however, the present study evaluates the approach specifically on Influenza A virus peptide sequences.

An additional design consideration concerns the selection of the specific protein language model configuration used in this study. The ESM-2 (facebook/esm2_t6_8M_UR50D) model was selected as the pretrained backbone due to its compact architecture and favorable balance between representational capability and computational efficiency. Larger variants of ESM-2, such as esm2_t33_650M_UR50D, contain substantially more parameters and therefore provide higher model capacity; however, they also impose significantly greater computational and memory requirements. For peptide-level prediction tasks involving short sequences, the marginal performance gains obtained from very large models may not justify the associated computational overhead. In contrast, the smaller ESM-2 configuration used in this work is capable of capturing meaningful contextual relationships among amino acid residues while enabling efficient training and inference. This balance between performance and efficiency is particularly important for practical immunoinformatics pipelines, where large-scale screening of peptide candidates is often required.

Interpretability remains a critical consideration in the application of DL models to immunological problems. The attention-based analysis conducted in this study provides insight into which residues and sequence regions influence epitope predictions. While attention weights should not be interpreted as direct evidence of biological causality, the concentration of attention on specific regions suggests that the model relies on structured sequence patterns rather than arbitrary correlations. For Influenza A virus, such regions may correspond to conserved or immunologically relevant segments that are repeatedly recognized by T cells, lending additional biological plausibility to the model’s behavior. This level of transparency is particularly valuable when computational predictions are used to guide experimental validation. Although the attention patterns provide useful qualitative insight into sequence regions that influence model predictions, a systematic comparison between attention-highlighted residues and experimentally characterized immunodominant epitopes of Influenza A virus would require detailed immunological and structural analyses across multiple datasets, which represents an important direction for future investigation.

The incorporation of uncertainty estimation through Monte Carlo dropout further enhances the practical relevance of the proposed framework. In influenza research, where experimental validation is costly and time-sensitive, incorrect high-confidence predictions can lead to inefficient use of resources. By associating each prediction with an uncertainty estimate, the model distinguishes high-confidence epitope candidates from ambiguous peptides that may require additional scrutiny. This capability aligns the computational pipeline more closely with real-world decision-making processes in vaccine research and immunological screening. It is worth noting that several recent sequence prediction studies employ pretrained protein language model embeddings followed by simple linear or shallow classification layers. While such approaches can achieve competitive performance, they often treat the PLM embedding as a fixed global representation without further modeling residue-level interactions relevant to the downstream task. In the proposed framework, the additional Transformer module serves as a task-specific refinement stage that operates on PLM-derived representations to capture positional relationships and interaction patterns within peptide sequences. This design allows the classifier to focus on sequence regions that contribute most strongly to epitope recognition, while maintaining a lightweight architecture that avoids the computational cost of full PLM fine-tuning. Consequently, the contribution of the present work lies in integrating contextual PLM representations with attention-based sequence modeling and uncertainty-aware prediction within a unified framework tailored for Influenza A T-cell epitope discovery.

Despite these strengths, several limitations should be acknowledged. The current framework operates exclusively at the sequence level and does not explicitly incorporate structural information, host MHC allele specificity, or broader immunological context, all of which influence T-cell recognition. Additionally, while freezing the protein language model improves stability, fine-tuning on larger and more diverse epitope datasets may further enhance task-specific performance. Another limitation is that the current framework focuses solely on peptide sequence information and does not explicitly incorporate host-specific MHC allele context, which plays an important role in T-cell recognition. In addition, the dataset used in this study is derived from curated experimental records in IEDB, and potential dataset biases or incomplete coverage of allele-specific immune responses may influence model generalization. Future work may therefore explore integrating allele-specific information, structural context, or broader immunological features to further enhance biological realism. Finally, this study focuses on Influenza A virus to enable controlled analysis; extending the framework to multiple viruses will require careful consideration of domain shifts arising from differing evolutionary pressures and sequence characteristics. Future extensions could integrate allele-specific binding information or peptide-MHC interaction modeling to further enhance biological realism. Future work may also explore: (a) more detailed calibration analyses of uncertainty estimates, including reliability diagrams or comparisons with alternative uncertainty quantification techniques, to further evaluate the reliability of predictive confidence measures. (b) evaluation on independent external datasets, as well as more detailed statistical and calibration analyses, to further assess the generalization and reliability of the proposed framework. In addition, although stratified cross-validation provides a robust internal evaluation strategy, future studies may further examine the generalization capability of the framework using independent datasets or homology-aware data partitioning strategies.

## 5. Conclusions

In this study, a transformer-based viroinformatics framework was presented for the prediction of TCEs from Influenza A virus peptide sequences. Influenza A virus is characterized by rapid genetic evolution and antigenic variability, which continue to challenge conventional vaccine design strategies. By integrating a pretrained PLM with a task-specific Transformer classifier, the proposed framework moves beyond traditional feature engineering and learns directly from raw amino acid sequences. This design enables the model to capture contextual and relational information that is difficult to represent using handcrafted physicochemical descriptors and is particularly relevant for modeling immune recognition in highly variable viral pathogens.

The findings demonstrate that protein language model-derived embeddings provide a strong representational foundation for epitope prediction, while the attention-based Transformer effectively learns epitope-specific sequence patterns from these embeddings. The framework exhibited stable and competitive performance across multiple evaluation settings, suggesting reliable generalization rather than dependence on favorable data partitions. The integration of uncertainty estimation further strengthens the practical utility of the model by enabling differentiation between high-confidence epitope predictions and ambiguous peptide candidates, an important consideration when computational outputs are used to guide experimental validation in influenza research.

Beyond predictive performance, the proposed framework offers interpretability through attention-based analysis, providing residue-level insights into sequence regions that influence epitope recognition. Although such explanations should be interpreted with caution, they contribute to increased transparency and help align model behavior with biological reasoning. Future work may explore the incorporation of structural information, host-specific context, or larger multi-virus datasets to further improve predictive accuracy and biological relevance.

## Figures and Tables

**Table 1:** Summary of the Influenza A virus peptide dataset used in this study.

Description	Value
Virus	Influenza A
Data source	IEDB
Total peptide sequences	8271
Positive samples (epitopes)	4136
Negative samples (non-epitopes)	4135
Class balance	~50:50
Peptide length range	8–15 amino acids
Task type	Binary classification

**Table 2:** Hyperparameter configuration of the proposed PLM–Transformer model.

Component	Setting
Protein Language Model	ESM-2 (facebook/esm2_t6_8M_UR50D) [33]
PLM embedding dimension	320
Transformer layers	1
Attention heads	4
Dropout rate	0.3
Optimizer	Adam
Learning rate	1 × 10^−4^
Batch size	16
Loss function	Binary cross-entropy
Training epochs	10

**Table 3:** Average performance of the proposed model obtained using 5-fold cross-validation.

Metric	Mean (%)	Std (%)
Accuracy	96.84	±0.42
Precision	96.11	±0.51
Recall (Sensitivity)	97.32	±0.38
Specificity	96.36	±0.44
F1-score	96.71	±0.40
AUC	0.989	±0.006
MCC	0.934	±0.008
Gini coefficient	0.978	±0.012

**Table 4:** Performance comparison between the proposed PLM–Transformer model and classical machine learning approaches.

Model	Accuracy (%)	F1-Score	AUC
Decision Tree [35]	88.42	0.882	0.912
SVM [36]	90.15	0.901	0.928
Random Forest [37]	92.73	0.926	0.947
Proposed PLM-Transformer	96.84	0.967	0.989

**Table 5:** Comparison of the proposed model with existing epitope prediction tools.

Method	Accuracy (%)	Sensitivity	Specificity
NetMHC [38]	85.34	0.861	0.842
CTLpred [39]	87.92	0.889	0.872
Proposed model	96.84	0.973	0.964

**Table 6:** Ablation analysis evaluating the contribution of the Protein Language Model (PLM) to overall model performance.

Model Variant	Feature Representation	Accuracy (%)	F1-Score	AUC	MCC
No-PLM + SVM	Physicochemical features	90.15	0.901	0.928	0.803
No-PLM + Transformer	Physicochemical features	92.48	0.924	0.944	0.849
PLM + Logistic Head	PLM embeddings only	94.72	0.947	0.967	0.894
PLM + Transformer (Proposed)	PLM embeddings + attention	96.84	0.967	0.989	0.934

**Table 7:** Effect of varying the number of attention heads on epitope prediction performance.

Attention Heads	Accuracy (%)	F1-Score	AUC	Remarks
1	94.96	0.949	0.972	Limited contextual modeling
2	95.88	0.958	0.981	Improved residue interactions
4	96.84	0.967	0.989	Best performance
8	96.79	0.966	0.988	Marginal gain, higher complexity

**Table 8:** Comparison of training time and computational cost across different modeling approaches.

Model	Feature Extraction Time	Training Time (per Epoch)	Total Parameters	Remarks
SVM (Physicochemical)	High	Low	~10^5^	Manual feature overhead
Random Forest	High	Moderate	~10^6^	Feature-dependent
CNN baseline	Low	High	~2.5 × 10^6^	Needs large data
PLM (Frozen) + Logistic	Very Low	Low	~8 M (frozen)	Efficient
PLM + Transformer (Proposed)	Very Low	Moderate	~8.4 M	Best accuracy–cost tradeoff

**Table 9:** Performance of the proposed model across different peptide length categories.

Peptide Length (aa)	Accuracy (%)	Sensitivity	Specificity	AUC
8–9	97.12	0.975	0.968	0.991
10–11	97.48	0.981	0.972	0.994
12–13	96.61	0.969	0.963	0.987
14–15	95.94	0.958	0.955	0.982

**Table 10:** Uncertainty estimation results using Monte Carlo dropout.

Prediction Category	Mean Probability	Mean Uncertainty
High-confidence epitopes	0.93	0.03
Borderline peptides	0.56	0.18
High-confidence non-epitopes	0.11	0.04

## Data Availability

The peptide data used in this study were obtained from the publicly available Immune Epitope Database (IEDB) (https://www.iedb.org (accessed on 22 December 2025)). The dataset can be reproduced by querying the IEDB database using the criteria described in the Materials and Methods section.

## Abbreviations

The following abbreviations are used in this manuscript:

TCE	T-cell Epitope
RNA	Ribonucleic Acid
MHC	Major Histocompatibility Complex
ML	Machine Learning
DL	Deep Learning
PLM	Protein Language Model
ESM-2	Evolutionary Scale Modeling-2
IEDB	Immune Epitope Database
ROC	Receiver Operating Characteristic
GPU	Graphics Processing Unit
